# Oxidative stress in cancer-bearing dogs assessed by measuring serum malondialdehyde

**DOI:** 10.1186/1746-6148-9-101

**Published:** 2013-05-11

**Authors:** Arayaporn Macotpet, Fanan Suksawat, Peerapol Sukon, Komgrit Pimpakdee, Ekkachai Pattarapanwichien, Roongpet Tangrassameeprasert, Patcharee Boonsiri

**Affiliations:** 1 Department of Medicine, Faculty of Veterinary Medicine, Khon Kaen University, Khon Kaen, 40002, Thailand; 2 Department of Anatomy, Faculty of Veterinary Medicine, Khon Kaen University, Khon Kaen, 40002, Thailand; 3 Department of Public Health, Faculty of Veterinary Medicine, Khon Kaen University, Khon Kaen, 40002, Thailand; 4 Department of Pathobiology, Faculty of Veterinary Medicine, Khon Kaen University, Khon Kaen, 40002, Thailand; 5 Department of Biochemistry, Faculty of Medicine, Khon Kaen University, Khon Kaen, 40002, Thailand

**Keywords:** Oxidative stress, Malondialdehyde, Cancer, Dog

## Abstract

**Background:**

Oxidative stress, an excess of reactive oxygen species (ROS), causes lipid peroxidation resulting in cell and tissue damages. It may be associated with the development and progression of cancers in dogs. Malondialdehyde (MDA), the end product of lipid peroxidation, is commonly used as a marker of oxidative stress. The objective of this study was to assess oxidative stress in cancer-bearing dogs by measuring serum MDA levels. All client-owned dogs underwent physical examination at the Veterinary Teaching Hospital, Faculty of Veterinary Medicine, Khon Kaen University to determine the health status with the owner’s consent. Blood samples of cancer-bearing dogs (N = 80) and clinically normal dogs (N = 101) were obtained and subjected for determination of MDA levels. In addition, complete blood count, creatinine, and alanine aminotransferase were measured.

**Results:**

Serum MDA was significantly higher in cancer-bearing dogs than in clinically normal dogs (mean ± SD, 4.68 ± 1.32 μmol/L vs 2.95 ± 0.61 μmol/L, respectively; p < 0.001). Packed cell volume (mean ± SD, 36.18 ± 7.65% vs 44.84 ± 5.54%), hemoglobin (mean ± SD, 11.93 ± 2.88 g% vs 15.17 ± 2.00 g%) and red blood cells (median (IQA), 6.05 (2.15) vs 8.09 (1.34)) were all significantly lower in cancer-bearing dogs than in clinically normal dogs. However, the reverse was true for white blood cells (median (IQA), 18.20 (11.95) vs 14.90 (5.10)). Neither creatinine nor alanine aminotransferase levels were significantly different between groups.

**Conclusions:**

This study supports the conclusion that oxidative stress is associated with many types of cancers in dogs, as serum MDA levels were significantly higher in cancer-bearing dogs compared to clinically normal dogs.

## Background

Oxidative stress is defined as an excess of reactive oxygen species (ROS) due to an imbalance between the rates of ROS production and ROS removal. Excess may be caused by the overproduction of ROS, the reduction of antioxidants that reduce ROS or both conditions [1]. ROS can seriously alter the structure of molecules such as proteins, lipids, and deoxyribonucleic acid (DNA) [2]. These alterations may result in cell degeneration and death causing aging [3], and play a significant role in the pathogenesis of many diseases, such as cardiovascular diseases, neuropathies, inflammatory diseases, acquired immune deficiency syndrome (AIDS), diabetes mellitus, renal diseases, and mammalian cancers [4-14]. In dogs, oxidative stress has been associated with carcinogenesis [15]. Cancer in dogs is a major cause of death [16-18] and in Thailand there appears to be an increase in the incidence of dog cancers, as the number of cancer-bearing dogs diagnosed at the Veterinary Teaching Hospital, Khon Kaen University, Thailand between January 2008 and December 2011, increased by more than 50% (data from the annual reports of the hospital).

Since oxidative stress triggers various antioxidant mechanisms in the body, biomarkers such as lipid peroxidation products and endogenous enzymes with antioxidant properties have been identified and used to assess oxidative stress in mammals [1]. Malondialdehyde (MDA), one of the end products of lipid peroxidation has been widely used as a biomarker of oxidative stress. MDA can be measured using several different assays; however, the simplest and most common method is the thiobarbituric acid reactive substances assay (TBARS) [19]. Although the use of MDA as a biomarker of oxidative stress in dogs is still controversial, a study on dogs with mammary gland tumors demonstrated that TBARS levels were significantly higher in the tumor tissue than in the normal tissue [20]. In contrast, in two other studies, plasma MDA levels in dogs with mammary tumors and with lymphomas did not differ from those in control dogs [15,21]. Reports from studies in humans indicated that serum and/or plasma MDA levels were elevated in association with various types of cancers (breast cancer [22,23], oral cancer [24], and lung cancer [22,25]).

As there are few studies on oxidative stress in cancer-bearing dogs compared to humans with cancer, the primary objective of this study was to evaluate oxidative stress status in cancer-bearing dogs compared to clinically normal dogs by measuring MDA levels.

## Methods

### Animals

Client-owned dogs were enrolled in this study between January 2010 and June 2011 at Veterinary Teaching Hospital, Faculty of Veterinary Medicine, Khon Kaen University, Khon Kaen, Thailand. Cancer-bearing dogs had to be diagnosed with a malignant tumor by histopathologists. The types of cancer were classified by evaluation histopathological slides from a tissue biopsy. Dogs undergoing chemotherapy or receiving antioxidant supplements were excluded. Clinically normal dogs had to be more than 2 years old without blood parasites or intestinal parasites and have had a normal physical examination at least 6 months before blood collection. Owner consent was obtained for all participated dogs and the study protocol was approved by The Research Ethics Committee, Faculty of Veterinary Medicine, Khon Kaen University.

### Blood sample collection, preparation and analysis

Five mLs of blood was drawn from the cephalic vein and divided into 2 parts. One mL was treated with ethylenediamine tetraacetic acid (EDTA), an anticoagulant and used to determine a complete blood cell count and the presence of blood parasites. The remainder was centrifuged at 3000 rpm at 4°C for 10 min to provide serum for blood chemistry tests (e.g. creatinine and alanine aminotransferase (ALT) and MDA).

Packed cell volume (PCV), hemoglobin, red blood cell counts (RBC) and white blood cell counts (WBC) were measured by using an automatic analyzer (Sysmex XT-2000iV, USA). Additionally, dried blood smears were fixed in methyl alcohol and stained with Wright-Giemsa stain and examined under oil immersion lens of the microscope for parasites. Creatinine and ALT were measured by using an automatic blood chemistry analyzer (Olympus AU400, USA).

### Determination of malondialdehyde (MDA)

MDA concentrations were determined using serum samples kept at −80°C and the thiobarbituric acid reactive substance assay (TBARS) [26,27]. One mL of serum diluted with normal saline, at a ratio of 1:1, was mixed with 50 μL of 0.1 mmol/L butylated hydroxytoluene, 500 μL of 5 mmol/L EDTA, 1 mL of 8% sodium dodecyl sulfate, 1 mL of 10% trichloroacetic acid and 1.5 mL of 0.67% thiobarbituric acid. The reaction mixture was incubated at 95°C for 30 min and then centrifuged at 3000 rpm at room temperature for 15 min. The absorbance of the supernatant was measured at 532 nm and tetraethoxypropane was used to prepare a standard curve at concentration ranges between 0.01- 0.2 μmol/L.

### Statistical analysis

The normality of continuous data was assessed using graphical display and the Shapiro-Wilk test. Data that met the parametric assumptions, non-parametric data, and categorical data were analyzed by an Independent Sample t-test, Mann–Whitney U test and Pearson Chi-square test, respectively. To account for the imbalance in age between cancer-bearing dogs and clinically normal dogs, age was used as a covariate in an Analysis of Covariance (ANCOVA) and an adjusted p-value was obtained. To determine whether MDA levels differed among cancer types, subgroup analysis was performed, as the number of different cancer types found in this study varied considerably. The less common cancers (i.e., squamous cell carcinoma, melanoma, lymphoma, adenocarcinoma, transmissible venereal tumor (malignant), hemangiosarcoma, and angiosarcoma) were combined and defined as “other cancers”. Data with normal distributions were reported as means ± SD, but those without normal distribution were reported as medians (interquartile range). All statistical analyses were performed by the statistical software (SPSS version 17; SPSS Inc., Chicago, Ill.) and values of p < 0.05 were considered significant.

## Results

Of the total 320 dogs, 107 were diagnosed with malignant tumors and 213 were identified as clinically normal dogs. Twenty-seven of the cancer-bearing dogs were excluded from the study, as they did not fulfill the inclusion criteria. Hence, 80 cancer-bearing dogs were used for final analysis. Of 213 clinically normal dogs, 112 dogs were excluded because they did not fulfill the inclusion criteria and therefore 101 clinically normal dogs were used in this study.

Demographic characteristics of the cancer-bearing and clinically normal dogs are shown in Table 1. Average age in cancer-bearing dogs was significantly higher than that in clinically normal dogs (mean ± SD, 9.06 ± 3.12 yrs vs 5.05 ± 2.20 yrs, respectively; p < 0.001); however, there was no significant difference in body weight between groups (p = 0.663). The proportion of pure breeds in the cancer-bearing dogs was significantly greater than that in the clinically normal dogs (47/80 or 58.75% vs 36/101 or 35.67%, respectively; p = 0.0032). In the cancer-bearing dogs, the pure breeds were Poodle (N = 10), Rottweiler (9), Thai Ridgeback (7), Cocker Spaniel (5), Golden Retriever (4), German Shepherd (2), Saint Bernard (2), Shih Tzu (2), Chow Chow (1), Dalmatian (1), Labrador Retriever (1), Miniature Pinscher (1), Pit Bull (1), and Siberian Husky (1). In the clinically normal dogs, the pure breeds were Golden Retriever (N = 8), Labrador Retriever (8), Thai Ridgeback (8), German Shepherd (3), Cocker Spaniel (2), Miniature Pinscher (1), Pit Bull (2), Pomeranian (1), Poodle (1), Rottweiler (1), and Siberian Husky (1). Although 10 types of cancers were found in this study, the three most common were mammary gland carcinoma (27/80 or 34%), mast cell tumor (15/80 or 19%), and osteosarcoma (8/80 or 10%) (Table 2).

**Table 1:** Demographic characteristic of the cancer-bearing dogs and clinically normal dogs

**Variables**	**Cancer-bearing dogs** **(N = 80)**	**Clinically normal dogs** **(N = 101)**	**p-value**^**a**^
Age, mean ± SD (range), yrs	9.06 ± 3.12 (2–15)	5.05 ± 2.20 (2–12)	<0.001
Weight, mean ± SD (range), kg	20.20 ± 11.87 (4.2-49.2)	20.89 ± 9.24 (4.8-40.4)	0.663
Breed, no. (%)			0.003
Mixed	33 (41.25%)	65 (64.36%)	
Pure breed	47 (58.75%)	36 (35.67%)	
Sex, no. (%)			0.428
Male	26 (32.50%)	38 (37.62%)	
Female	54 (67.50%)	63 (62.38%)	

^a^Age and weight by Independent Sample t-test; breed by Chi-square test.

**Table 2:** Number and percentage of dogs classified with different cancer types

**Cancer type**	**Number of dogs (%)**
Mammary gland carcinoma	27 (34%)
Mast cell tumors	15 (19%)
Osteosarcoma	8 (10%)
Squamous cell carcinoma	6 (7%)
Lymphoma	5 (6%)
Melanoma	5 (6%)
Fibrosarcoma	5 (6%)
Adenocarcinoma	4 (5%)
Malignant TVT	3 (4%)
Hemangiosarcoma	2 (3%)

**Figure 1:**
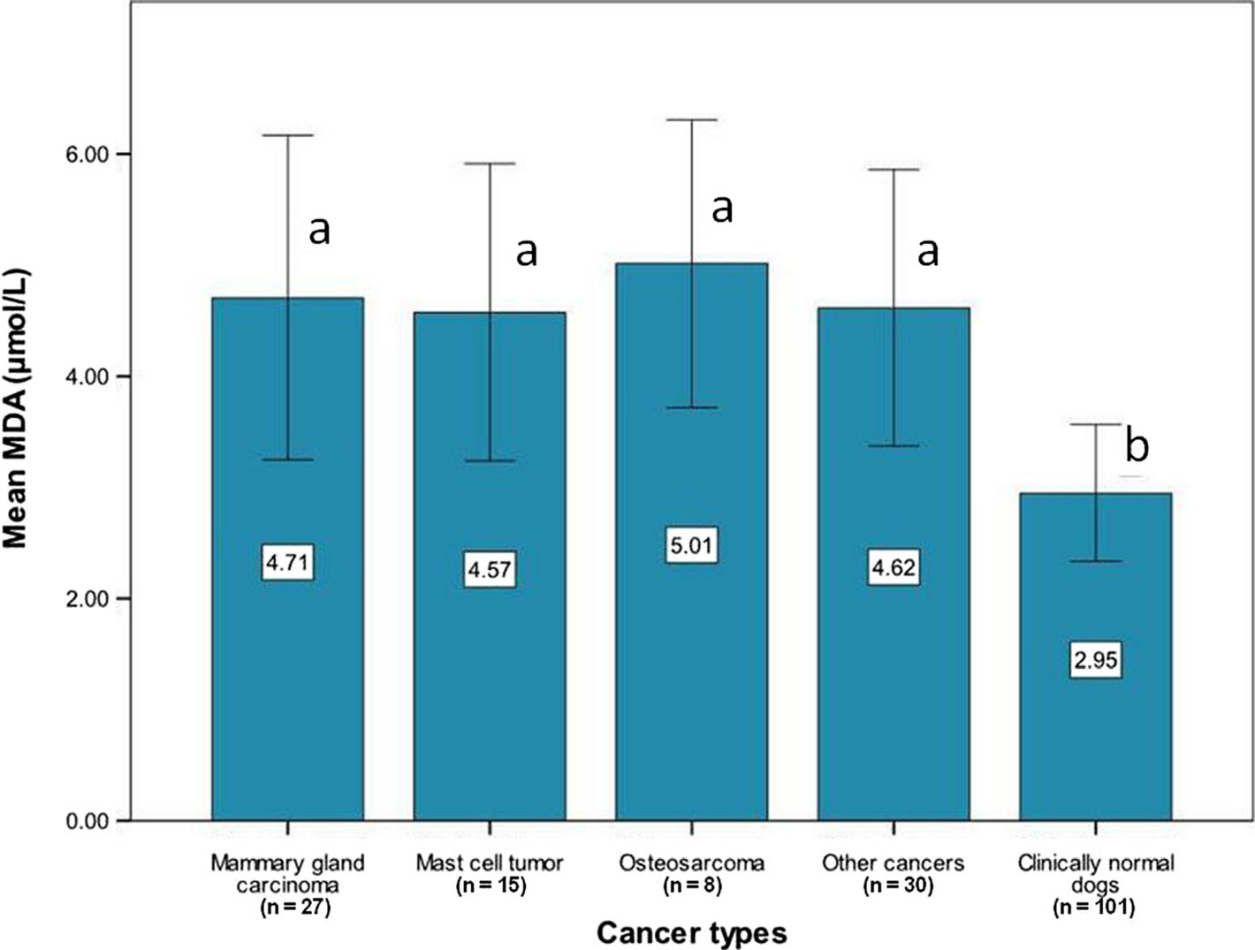
**Mean MDA levels in dogs with different cancer types and clinically normal dogs.** Means with the same letter (a or b) are not significantly different and error bars indicate standard deviations.

Serum MDA was significantly higher in cancer-bearing dogs than in clinically normal dogs even with age adjusted (mean ± SD, 4.68 ± 1.32 μmol/L vs 2.95 ± 0.61 μmol/L, respectively; p < 0.001) (Table 3) and serum MDA did not differ significantly among cancer types (p = 0.826) (Figure 1).

**Table 3:** Malondialdehyde, complete blood counts and blood chemistry parameters for cancer-bearing dogs and clinically normal dogs

**Variables**	**Cancer-bearing dogs** **(N = 80)**	**Clinically normal dogs** **(N = 101)**	**p-value**^**a**^	**Adjusted p-value**^**b**^	**Normal ranges**[28]
MDA, mean ± SD, μmol/L	4.68 ± 1.32	2.95 ± 0.61	<0.001	<0.001	
PCV, mean ± SD, %	36.18 ± 7.65	44.84 ± 5.54	<0.001	<0.001	37-55
Hemoglobin, mean ± SD, g%	11.93 ± 2.88	15.17 ± 2.00	<0.001	<0.001	12-18
RBC, median (IQA), 10^6^/μL	6.05 (2.15)	8.09 (1.34)	<0.001	NA	5.5-8.5
WBC, median (IQA), 10^3^/μL	18.20 (11.95)	14.90 (5.10)	<0.001	NA	6-17
Creatinine, median (IQA), mg/dL	0.90 (0.30)	1.00 (0.25)	0.312	NA	0.9-1.7
ALT, median (IQA), U/L	32.00 (33.00)	36.00 (26.00)	0.395	NA	10-120

Abbreviations: *ALT* alanine aminotransferase, *MDA* malondialdehyde, *NA* not applicable, *PCV* packed cell volume, *IQA* interquartile range, *RBC* red blood cells, *SD* standard deviation, *U/L* unit per liter, *WBC* white blood cells.

^a^An independent sample Students t-test was used for MDA, PCV, and hemoglobin, while a Mann–Whitney U test was used for RBC, WBC, creatinine, and ALT.

^b^Adjusted p-values were obtained from an analysis of covariance using the age of dogs as a covariate.

The number of red blood cells and white blood cells in both groups remain within normal ranges [28] (Table 3); however, red blood cell counts were significantly lower in cancer-bearing dogs than in clinically normal dogs (p < 0.001), whereas white blood cell counts were significantly higher in cancer-bearing dogs (p < 0.001) (Table 3). No blood parasites were found and creatinine and ALT levels were within normal ranges and not significantly different between the two groups (p = 0.312 and p = 0.395, respectively).

## Discussion

As the design of this study is cross-sectional, the inference cannot be drawn that the oxidative stress causes cancers or vice versa. Ideally, the best way to assess the relationship between oxidative stress and ROS is to measure ROS directly. However, ROS are difficult to measure with standard biochemical techniques due to their high reactivity and short half-life. Therefore, surrogate markers are required and MDA is commonly used as a biomarker of oxidative stress for various pathological conditions and diseases, including cancers [15,24,29,30].

In the present study, it was clear that MDA levels were significantly higher in cancer-bearing dogs compared to clinically normal dogs (Table 3). Moreover, MDA was significantly higher in all cancer types studied in the subgroup analysis (Figure 1). These results correspond to previous studies on humans with breast cancer [22,23,29], oral cancer [24] and lung cancer [22,25]. However, controversy still exists for the use of MDA as a biomarker for oxidative stress in cancer-bearing dogs, because MDA values tend to vary greatly among laboratories [15,23,24,29], which may be related to the use of different types of MDA analyses and/or experimental design [30].

A few studies report that MDA levels in dogs with mammary gland tumors and lymphomas were not significantly different than those in control dogs [15]. However, other studies on dogs with mammary tumors reported that TBARS levels in tumor tissues were significantly higher than those in the normal tissue [20] and concluded that increased levels of MDA in dogs with pathological conditions was associated with oxidative stress [31,32].

In the present study, the average age of dogs with cancer (9 years old) was significantly greater than the control group (5 years old) (Table 1), which potentially could explain differences in MDA levels. However, the correlation analysis demonstrated that age was not correlated with MDA levels in either cancer-bearing dogs or clinically normal dogs and MDA levels did not changed with age. To further confirm that differences in age did not contribute to differences in MDA levels, we did an additional analysis by excluding dogs aged 7 years or higher in both groups. In this analysis, we found that age was not significant different in either groups (mean ± SD, 4.43 ± 1.34 yrs in cancer-bearing dogs (N = 14) vs 4.07 ± 1.33 yrs in clinically normal dogs (N = 76)), but serum MDA levels were still significantly higher in cancer-bearing dogs (mean ± SD, 4.85 ± 1.62 μmol/L vs 2.92 ± 0.59 μmol/L, respectively; p < 0.001). It is theorized that most cancers in older dogs is due to an increase in free radicals with age and free radical invasion of DNA over a long period. This results in DNA damage, mutations, and possibly carcinogenesis [33-36] and ultimately the loss of cell function and cell death [37].

In this study, red blood cell counts in cancer-bearing dogs were lower than those of healthy dogs, although they remained within the normal range. As anemia is one of the most common para-neoplastic syndromes [38,39] and may develop with a malignancy through various mechanism [40], we conclude that cancer-bearing dogs have tendency to be anemic. In contrast, white blood cell counts of cancer-bearing dogs were higher than those of healthy dogs, which may be caused by tissue damage [41], inflammation [42], oxidative stress [8], stress [43] and concomitant infections [44] (Table 3).

## Conclusions

In summary, this study supports the conclusions; (1) that oxidative stress is associated with many types of cancers in dogs, as serum MDA levels were significantly higher in cancer-bearing dogs compared to clinically normal dogs, (2) cancer-bearing dogs have a tendency towards anemia with significant lower levels of PCV and hemoglobin, (3) cancer-bearing dogs have elevated levels of white blood cells.

## Abbreviations

ALT: Alanine aminotransferase; kg: Kilogram; MDA: Malondialdehyde; mL: Milliliter; PCV: Packed cell volume; RBC: Red blood cell; TBARS: Thiobarbituric acid reactive substances; WBC: White blood cell; U/L: Unit per liter; Yrs: Years.

## Competing interests

None of the authors of this article have financial or personal relationships with other people or organizations that could bias the content of the manuscript.

## Authors’ contributions

AM, FS, PS, KP, and PB designed the study. AM, FS, and EP examined the health status of the dogs. EP identified the types of cancer. AM, RT, and PB measured MDA and other laboratory variables. PS performed the statistical analysis. AM, FS, PS, and PB drafted the manuscript and all authors read and approved the final submission.
